# Sunitinib-eluting thin films for Inhibition of corneal neovascularization

**DOI:** 10.1007/s13346-025-01926-5

**Published:** 2025-07-28

**Authors:** Kunal S. Parikh, Jin Yang, Zheng Ding, Richard Shi, Sagun Poudel, Yumin Oh, Lixia Luo, Shiyu Xia, Gregg Duncan, Charles Eberhart, Laura M. Ensign, Justin Hanes, Qingguo Xu

**Affiliations:** 1 Center for Nanomedicine, The Wilmer Eye Institute, Johns Hopkins University School of Medicine, Baltimore, MD 21231, USA; 2 Department of Ophthalmology, The Wilmer Eye Institute, Johns Hopkins University School of Medicine, Baltimore, MD 21287, USA; 3 Center for Bioengineering Innovation & Design, Johns Hopkins University, Baltimore, MD 21218, USA; 4 Department of Biomedical Engineering, Johns Hopkins University School of Medicine, Baltimore, MD 21205, USA; 5 Department of Ophthalmology, Myopia Key Laboratory of Health PR, Eye & ENT Hospital of Fudan University, Shanghai 200031, China; 6 Departments of Pharmaceutics, Virginia Commonwealth University, Richmond, VA 23298, USA; 7 State Key Laboratory of Ophthalmology, Zhongshan Ophthalmic Center, Sun Yat- sen University, Guangzhou 510060, China; 8 Department of Chemical and Biomolecular Engineering, The Johns Hopkins University, Baltimore, MD 21218, USA; 9 Fischell Department of Bioengineering, University of Maryland, College Park, MD 20742, USA; 10 Department of Pathology, The Johns Hopkins University School of Medicine, Baltimore, MD 21231, USA; 11 Departments of Ophthalmology, Pediatrics, Biomedical Engineering, Massey Cancer Center, Center for Pharmaceutical Engineering, Center for Drug Discovery, Virginia Commonwealth University, Richmond, VA 23298, USA

**Keywords:** Electrospinning, Sunitinib, Nanofiber, Corneal neovascularization, Tyrosine kinase inhibitor, Ocular drug delivery

## Abstract

Corneal disease is a leading cause of blindness globally, and is commonly associated with corneal neovascularization, which results in the loss of optical clarity and visual impairment. A wide variety of factors contribute to abnormal growth of new blood vessels into the cornea, including contact lens wear, infectious keratitis, corneal graft rejection, inflammatory disorders and trauma. In each case, proangiogenic factors such as vascular endothelial, platelet-derived, and fibroblast growth factors fuel new blood vessel growth and proliferation. Topical corticosteroids are a front-line treatment for corneal neovascularization, but require patient compliance and long-term use, which can limit efficacy and lead to increased intraocular pressure or cataract development, respectively. There is a need for a simple, effective treatment for corneal neovascularization that improves access and alleviates compliance issues. Here, biodegradable, sunitinib-loaded, nano- and micro-structured thin films were manufactured via electrospinning and applied topically in a rat model of corneal neovascularization. Thin films were characterized for morphology, mechanical and physicochemical properties, and drug release prior to in vivo assessment of biocompatibility and efficacy. Nano-structured thin films demonstrated sustained in vitro release of sunitinib for 30 days, provided sufficient strength for extended topical application, and were biocompatible on rat eyes. Importantly, sunitinib-loaded thin films inhibited corneal neovascularization more significantly than 3x daily sunitinib eye drops, as evidenced by greater reductions in vessel area, length, and vascular endothelial growth factor expression. This sustained drug-delivery platform has potential to provide a safe and effective treatment for ocular surface and corneal disorders.

## Introduction

Blood vessel infiltration into the normally avascular cornea, leading to edema, scarring, and inflammation, is a leading cause of visual impairment and blindness globally. It is estimated that 1.4 million individuals are affected by corneal neovascularization annually, leading to vision loss in more than 160,000 people [1–3]. Corneal neovascularization results from disorders of the cornea or insults to the cornea, including degeneration, ischemia, inflammation, infection, or trauma, that promote an imbalance of angiogenic factors such as vascular endothelial growth factor (VEGF), platelet-derived growth factor (PDGF), pigment epithelium derived factor, angiostatin, and endostatin [4–6]. Notably, neovascularization may also increase the risk of failure of ocular surgery. It has been reported that 20% of corneal specimens from penetrating keratoplasty procedures demonstrate neovascularization, with the risk of graft rejection increasing with the vascularization of the cornea [2, 7].

To date, treatments for corneal neovascularization are limited, particularly in severe cases. Topical corticosteroids are the accepted first-line treatment for corneal neovascularization, but they require patient compliance and long-term treatment in order to be effective, which may lead to glaucoma or cataract development [7]. Alternatively, therapeutic options such as anti-VEGF medications are expensive, have short half-lives, and have limited topical bioavailability. Notably, both options are limited in their capacity to treat already formed, mature vessels. Failure or exclusion of these treatments leads to use of laser and fine needle diathermy which are more invasive, may damage the cornea and lens, and have high re-operation rates due to reopening of the vessels [7]. New treatment modalities have been reported in the literature and in clinical trials to overcome the limitations of conventional approaches, including eye drops with greater residence time, drug-eluting contact lenses, gene therapy, and nanomedicine injections; however, they continue to suffer from limitations including the requirement for patient compliance, potential to achieve long-term release, or ability to terminate the treatment [8, 9]. Importantly, the price of these treatments may limit access in low resource settings which disproportionately experience the burden of corneal disease [10].

Here, we sought to develop a simple, effective, biocompatible platform for prevention of corneal neovascularization via local, sustained drug delivery. Thin films were manufactured via electrospinning, a versatile fabrication method that allows for manufacture of fibers composed of a wide range of polymers and therapeutic or prophylactic agents. Notably, electrospun fiber diameter can be tuned to modify film properties, and nanofibers have a high surface area to volume ratio, enabling increased drug loading and controlled drug release [11]. Thin films were formulated with poly(lactic-co-glycolic acid) (PLGA) and sunitinib in order to provide a highly effective anti-angiogenic modality. PLGA is a biodegradable, generally regarded as safe (GRAS) polymer that has been used extensively in drug delivery systems and medical devices, including sutures used in ophthalmology [12–14]. Sunitinib is a multi-targeted tyrosine kinase inhibitor that has been shown to suppress HIF-1α, and inhibit both vascular endothelial growth factor and platelet-derived growth factor receptors. However, hydrophobic small molecules such as sunitinib face barriers to corneal penetration and require frequent administration for efficacy [15, 16]. This study introduces the first sunitinib-loaded electrospun thin film applied topically for corneal neovascularization, offering a biodegradable, sustained delivery alternative to current approaches. These PLGA/sunitinib thin films were evaluated for biocompatibility and efficacy in a suture-induced rat model of corneal neovascularization.

## Materials and methods

### Materials

Poly(D, L-lactic-co-glycolic acid) (PLGA; 50:50; i.v. = 0.32–0.44 dl/g) was purchased from Evonik (Germany). Hexafluoroisopropanol (HFIP) was purchased from Sigma Aldrich (St. Louis, MO). Sunitinib malate (> 99%) was purchased from LC Laboratories (Woburn, MA). Disposable syringes, 1x Dulbecco’s Phosphate-Buffered Saline (PBS), a High Capacity cDNA Reverse Transcription Kit, and TRIzol^®^ were purchased from Thermo Fisher Scientific (Waltham, MA). 20 and 25 G blunt tip needles were purchased from Nordson EFD (East Providence, RI). Dimethyl sulfoxide (DMSO), acetonitrile, water and ethanol at analytical grade or above were purchased from Sigma Aldrich (St. Louis, MO). 10 – 0 nylon sutures were purchased from Alcon Laboratories (Fort Worth, TX).

### Fabrication of nano- and micro-fiber sunitinib thin films

To prepare nanofiber thin films, PLGA was dissolved in HFIP at 20% w/w and then electrospun using a custom-built electrospinning system for 120 min at a flow rate of 250 μL/hr through a 25 G blunt tip needle, with an applied voltage of 10.5 kV at a distance of 24 cm from a grounded collector [17–22]. PLGA/sunitinib thin films were manufactured under the same conditions at a 2% sunitinib malate and 20% PLGA concentration (w/w).

The weight% of PLGA was increased to 30% in HFIP to increase fiber diameter. To prepare microfiber thin films, PLGA was dissolved in HFIP and then electrospun for 20 min at a flow rate of 1 mL/hr through a 20 G blunt tip needle, with an applied voltage of 6.5 kV, and at a distance of 13 cm from a grounded collector. Microfiber PLGA/sunitinib thin films were manufactured similarly at a 3% sunitinib and 30% PLGA concentration (w/w).

### Morphological analysis

Nanofiber thin film morphology was examined using scanning electron microscopy (SEM). Thin films were sputter coated with 10 nm of Au/Pd prior to imaging at 1.5 kV on a LEO Field Emission SEM (Zeiss, Germany). Thin film thickness (*n* = 3, each) was measured via reflectance imaging on an LSM 510 Meta Confocal Microscope (Zeiss).

### Tensile strength measurement

Thin film breaking strength was evaluated using a DMA 6800 (TA Instruments, Timonium, MD). PLGA and PLGA/sunitinib thin films (*n* = 5, each) were cut to 12 mm in width, clamped vertically at a length of 4.3 mm, and stretched at a force of 0.6 N/min until breaking.

### Analysis of thin film permeability

Water vapor flux was used as a surrogate for corneal oxygen permeability and surface hydration. Thin film permeability was analyzed using Elcometer 5100/1 Payne permeability cups with a surface area of 10 cm^2^ (Elcometer, Inc., Rochester Hills, Michigan). The cups were filled with 5 mL of ultrapure water after which the PLGA and PLGA/sunitinib films (*n* = 3, each) were fitted to the top of the cup. The cup was then weighed and placed in a desiccator at 37 °C for the duration of the experiment. The weight of each cup was measured every hour for 5 h and then at 24 h to determine permeability.

### Drug loading and in vitro drug release

Sunitinib thin films were weighed and dissolved in DMSO. The solution was measured by UV-Vis at 441 nm on a BioTek Microplate Reader (Winooski, VT). The sunitinib drug concentration was calculated using a standard curve of sunitinib malate in DMSO. The drug loading (DL) and encapsulation efficiency (EE) were calculated as follows:

$$DL\left(\%\right)=\frac{amount\;of\;\text{Sunitinib}\;\text{malate}\;in\;thin\;film}{weight\;of\;thin\;film}$$

$$EE\left(\%\right)=\frac{actual\;drug\;loading}{theoretical\;drug\;loading}$$

To study the in vitro drug release profile of sunitinib thin films, 1–2 mg thin films were weighed and placed in 1.5 mL siliconized tubes. Tubes were placed on an orbital shaker in a 37 °C incubator and shaken at 120 rpm. At predetermined time points, the release media was collected and replaced with 1 mL fresh PBS. The concentration of sunitinib malate in the collected release media was measured by UV-Vis and calculated using a standard curve for sunitinib malate in PBS.

### Animals

All animals were cared for in accordance with protocols approved by the Johns Hopkins University Animal Care and Use Committee, the ARVO Statement for the Use of Animals in Ophthalmic and Vision Research, and the National Institutes of Health guide for the care and use of laboratory animals. Male Sprague Dawley rats (6–8 weeks old) were purchased from Harlan (Indianapolis, IN). The animals were anesthetized with intramuscular injection of a mixture of ketamine (50 mg/kg) and xylazine (5 mg/kg) during experimental procedures. Topical instillation of 0.5% proparacaine and 0.5% tropicamide were used for topical anesthesia and pupil dilation, respectively. Procedures were conducted only on one eye in each rat.

### Assessment of thin film biocompatibility

A 1 cm x 1 cm thin film (weight ~ 1.7–1.8 mg) was placed on the corneal surface and retained via four 10 – 0 silk suture stitches to the conjunctiva. An Elizabeth cone was attached after thin film placement to prevent removal by the rats. The animals had free access to food and water during the studies. For the biocompatibility study, films were placed for 7 days after which the thin films were removed under anesthesia and eyes imaged under slit lamp after fluorescein staining. Rats were then euthanized and eyes enucleated, fixed with 10% formalin, and embedded in paraffin. The paraffin sections were cut through the papillary optic nerve plane and stained with hematoxylin and eosin (H&E) for histological examination. Sections were examined by a masked pathologist.

### Evaluation of corneal neovascularization Inhibition

Corneal neovascularization was induced by intrastromal suturing, as described previously [23, 24]. In brief, rats were anesthetized, and their pupils were dilated prior to placing two intrastromal 10 – 0 nylon suture stitches in the superior cornea under an operating microscope. The distance between the stitches and the limbus was approximately 2 mm and there was a distance of 1 mm between the two stitches. After suturing, animals were randomly assigned to four treatment groups: (1) topical instillation of PBS (10 μL, 3 times per day), (2) topical instillation of sunitinib malate free drug solution (5 mg/mL, 10 μL, 3 times per day), (3) topical application of placebo thin film (1 cm x 1 cm, weight ~ 1.7–1.8 mg, 1 time) and (4) topical application of sunitinib-eluting thin film (1 cm x 1 cm, weight ~ 1.7–1.8 mg, containing ~ 0.15 mg Sunitinib, 1 time), as shown in Fig. S1A. An Elizabeth cone was attached after thin film placement for rats receiving topical application of placebo and sunitinib-eluting thin films (Fig. S1B). Each group was comprised of *n* = 6 rats for a total of 6 experimental eyes. The rats were evaluated for 7 days. Afterwards, the corneas of all rats were examined by slit-lamp biomicroscope (SL120; Carl Zeiss AG, Oberkochen, Germany) and corneal photographs were taken with a digital camera by an ophthalmic photographer in a masked manner. The area and length of vascularized cornea were quantified with ImageJ software using previously described methods [23, 24]. An arc was drawn along the limbus, and the corneal neovascularization (NV) area was calculated using the following equation:

$$Corneal\;NV\;area=\frac{\text{pixel}\;\text{of}\;\text{vascularized}\;\text{area}}{pixel\;to\;occupy\;1\;mm^{2}\;area}$$

The vascularized area was evenly divided into six sections. The distance between vessel tips and the limbus at the five intersection points of the arc was measured. The five measured lengths were averaged to calculate the corneal NV length. These measurements were carried out by masked graders. At the endpoint of 7 days after treatment, three rats were sacrificed and the eyeballs were enucleated and processed for histological examination, as described before. Sections were examined by a masked pathologist. The other three rats in each group were euthanized and the corneas were collected for qPCR. Because of the limited amount of corneal tissue from individual eyes, three corneas of the same condition were pooled together to collect sufficient tissue for mRNA isolation and measurements. The mRNA expression levels of angiogenic and anti-angiogenic factors including VEGF, VEGFR1, VEGFR2, PDGFRα, PDGFRβ, VE-cadherin, Ang1, MMP2, MMP9 were quantified by RT-PCR with Fast SYBR^®^ Green Master Mix using a 7100 Real Time PCR System (Applied Biosystems, CA). The primers are listed in Table S1. The mRNA expression levels were normalized to GAPDH. Each sample was repeated 3 times for the mRNA expression level quantification.

### Statistical analysis

Thin film thickness, strength, permeability, and in vitro drug release are presented as mean ± standard error. Animal study data are presented as the average ± standard error of the mean (SEM). Two groups were compared using two-tailed Student’s t-test and three or more groups were compared using one-way ANOVA followed by Tukey’s post-hoc test. Differences were considered to be statistically significant at a level of *p* < 0.05. Significance for multiple comparisons: *p < 0.05; **p < 0.01; ***p < 0.001.

## Results

We designed a sunitinib-loaded thin film for sustained prevention or treatment of corneal neovascularization via topical ocular administration in applications such as penetrating keratoplasty, in which the risk of graft failure is doubled by corneal neovascularization [5]. In order to provide convenience while also preventing side effects and precluding corneal neovascularization, the thin film must be biocompatible, biodegradable, provide significant and sustained drug release, allow for diffusion of oxygen to the eye, and have sufficient strength for topical application. We hypothesized that thin films composed of PLGA/sunitinib fibers would provide strength and permeability at the corneal surface for an extended duration while also delivering an anti-angiogenic agent in a sustained and controlled manner to prevent corneal neovascularization.

### Characterization of nano- and micro-fiber thin film

**Fig. 1:**
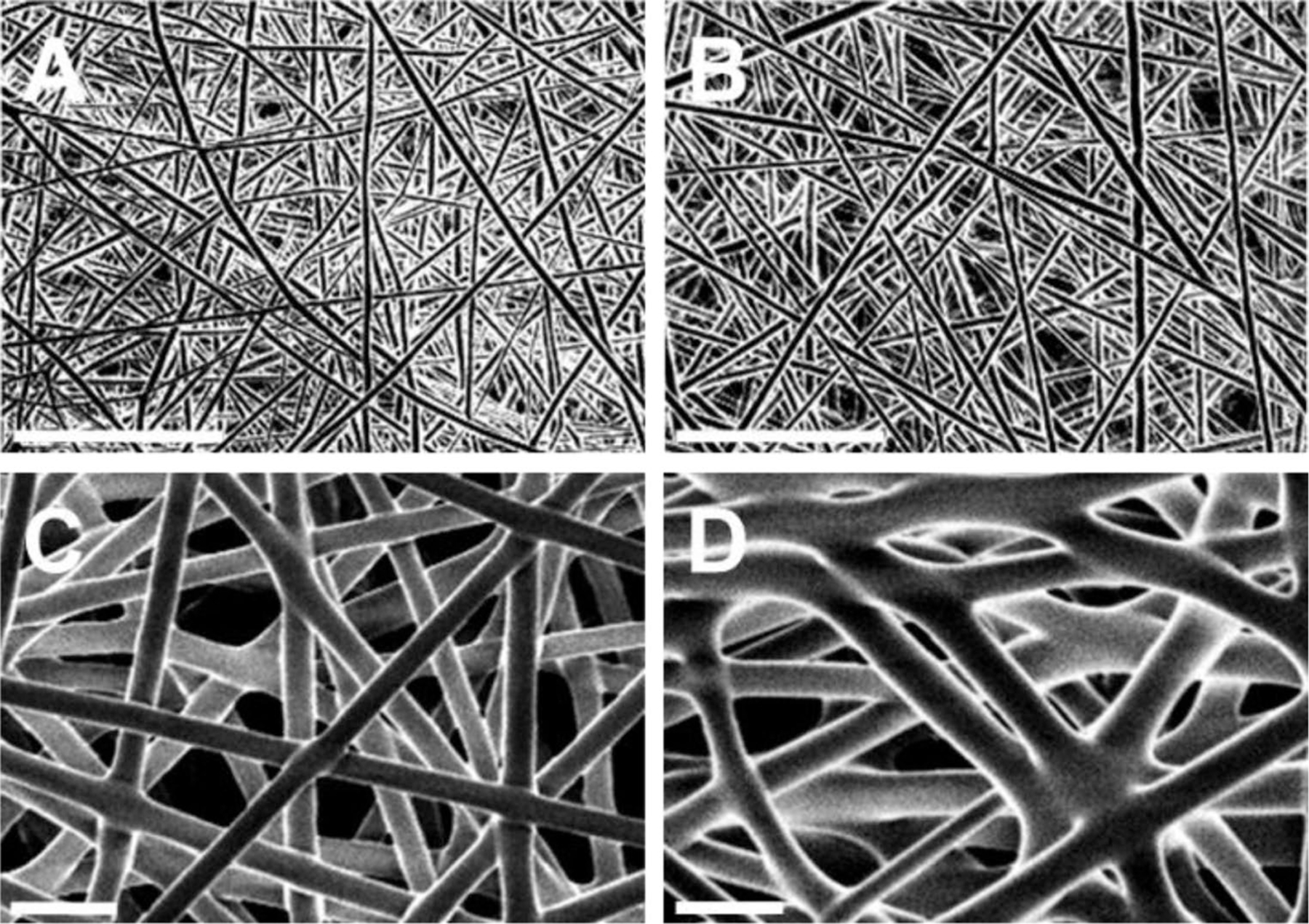
Representative SEM images of thin films composed of **(A)** PLGA nanofibers, **(B)** PLGA/sunitinib nanofibers, **(C)** PLGA microfibers, and **(D)** PLGA/sunitinib microfibers. Scale bars represent 10 μm

Sunitinib thin films were formulated via electrospinning of PLGA and PLGA/sunitinib solutions onto a flat, grounded collector. Solution formulations and electrospinning conditions were varied in order to reproducibly manufacture thin films composed of either nano- or micro-fibers. The nanofiber thin film formulation was composed of 20% PLGA in HFIP (w/w) and was manufactured by applying 10.5 kv to the polymer solution pumped at 250 μL/hr for 120 min towards the collector 24 cm away. The microfiber thin film formulation was composed of 30% PLGA in HFIP (w/w) and was manufactured by applying 6.5 kv to the polymer solution pumped at 1 mL/hr for 20 min towards the collector 13 cm away. Films were composed of porous layers of highly uniform nano- and microfibers, as shown in Fig. 1. The strength, permeability, and thickness of the films are listed in Table 1. Microfiber thin films (43–46 μm) were thicker than nanofiber films (13 μm) and provided greater tensile strength (1.04–1.08 N) in comparison to nanofiber thin films (0.64–0.69 N). However, microfiber films demonstrated greater permeability (214–218 (g/(h·m^2^))), as measured by benchtop water vapor flux measurements, than nanofiber films (175–186 (g/(h·m^2^))). The extremely thin nature of these electrospun films (13 μm in average thickness) provided for more permeability than would be expected from a thicker or non-porous film, while also providing sufficient strength for topical application [25, 26]. Notably, film characteristics, including strength, were similar for films with and without sunitinib.

### Drug loading and in vitro drug release

**Fig. 2:**
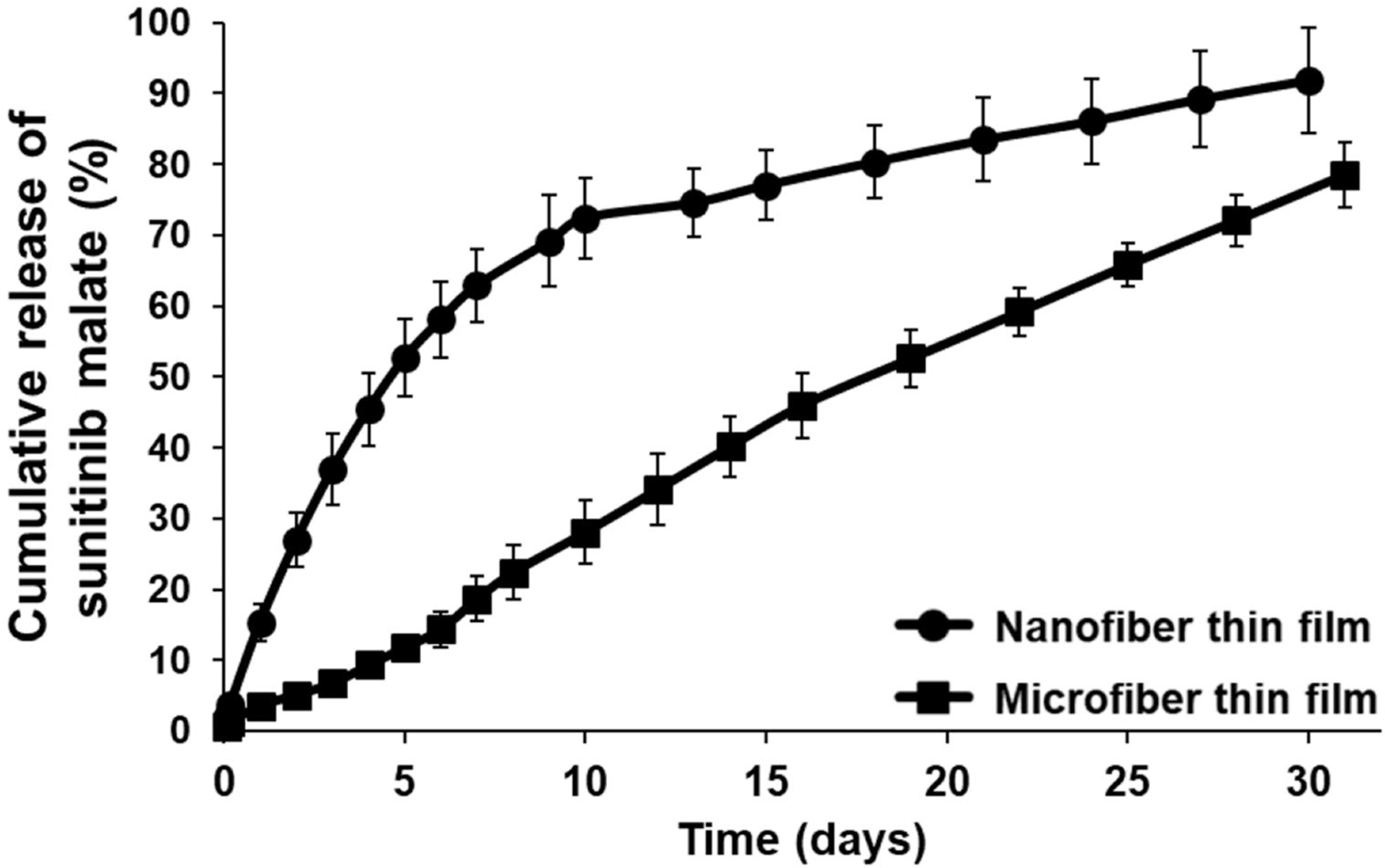
Cumulative release percentage of sunitinib from PLGA thin films composed of nano- or micro-fibers. Both formulations provided sustained release of sunitinib for at least four weeks

Thin films dissolved in DMSO immediately following fabrication revealed sunitinib loading of 8.6 ± 0.2 wt% and 7.6 ± 0.3 wt% in nano- and micro-fiber films, respectively. Both thin films demonstrated sustained in vitro sunitinib release for at least four weeks; however, there were marked differences in release profiles (Fig. 2). Sunitinib-loaded, nanofiber-based thin films demonstrated a two-phase drug release profile with an initial phase of rapid drug release during the first 10 days followed by slower release afterwards. Approximately 70% of total loaded sunitinib was released during the first 10 days followed by an additional 20% released in the following 20 days. Microfiber-based thin films demonstrated a linear drug release profile for at least 30 days. Only 25% of total loaded sunitinib was released during the first 10 days. This may be attributed to the increased thickness (46 ± 5 μm) and increased fiber diameter (4.5 ± 0.4 μm) of sunitinib-loaded microfiber films in comparison to sunitinib-loaded nanofiber films (13 ± 2 μm; 350 ± 30 nm). The nanofiber thin film formulation was chosen for animal efficacy studies due to its potential for increased drug delivery in the early post-operative period (~ 70% drug release in 10 days).

### Sunitinib nanofiber thin film is biocompatible

**Fig. 3:**
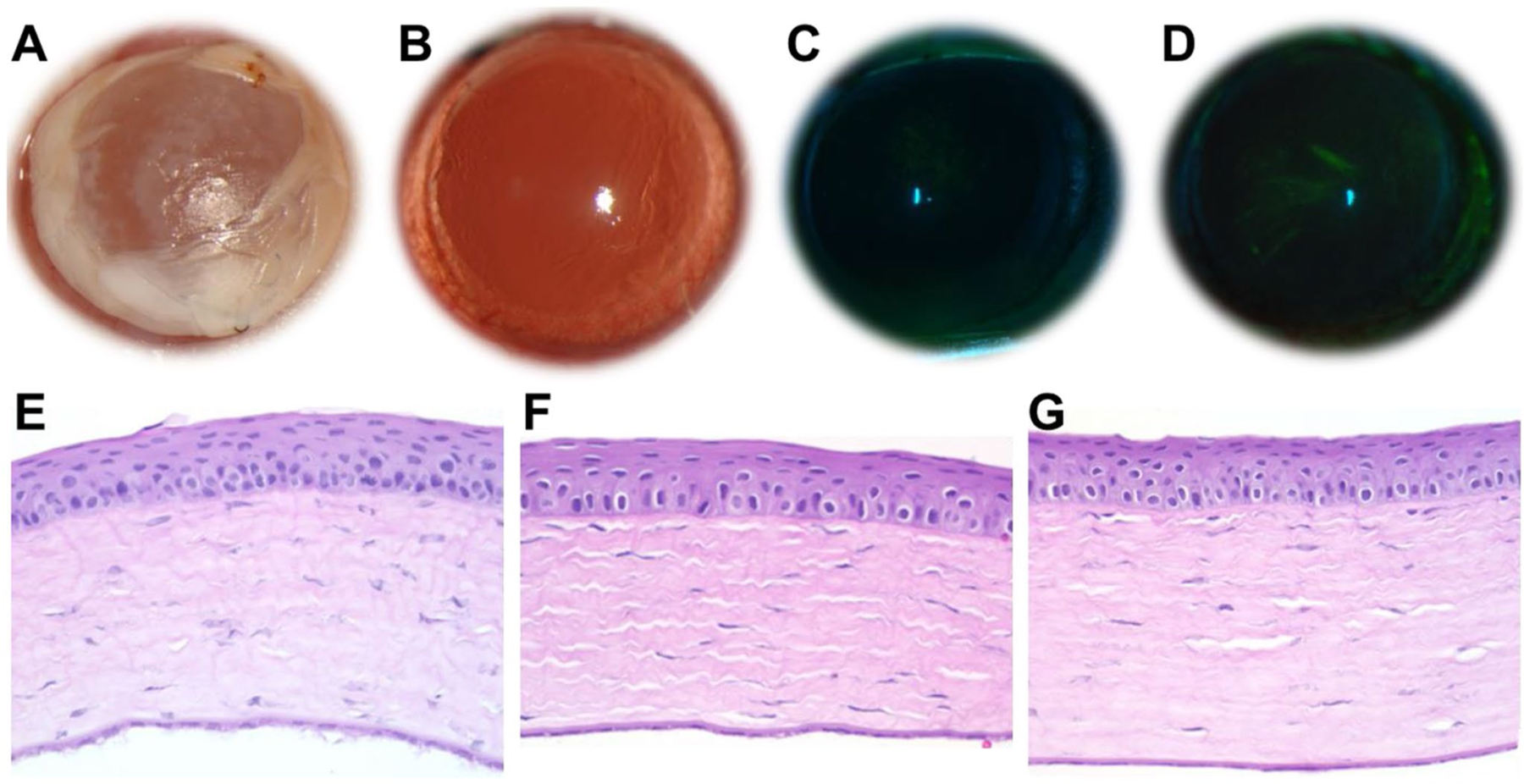
Representative images of rat eyes following application of **(A)** nanofiber-based, sunitinib-eluting thin film for 7 days, **(B)** immediately following removal of sunitinib-eluting thin film after 7 days, **(C)** healthy, contralateral eye following fluorescein staining, and **(D)** treated eye following fluorescein staining after removal of sunitinibeluting thin film after 7 days. Representative images of H&E staining of cross-sections of the cornea of a **(E)** healthy, contralateral eye, **(F)** eye receiving a placebo nanofiber thin film for 7 days, and **(G)** eye receiving a sunitinib-eluting nanofiber thin film for 7 days. No signs of irritation, inflammation, epithelial disruption, neovascularization, or toxicity were observed upon gross or histological assessment. Magnification for E-G: 200x

Nanofiber thin films with or without sunitinib were applied to Sprague Dawley rat corneas for 7 days via suturing through the conjunctiva. Films remained in place and were well-tolerated throughout the study period. There were no cases of inflammation, hyphema, infection or corneal neovascularization upon clinical exam, indicating that the films were biocompatible and provided sufficient oxygen diffusion (Fig. 3A–B). Notably, fluorescein staining of rat eyes receiving the thin film demonstrated no epithelial damage, and no significant differences in comparison to healthy control eyes (Fig. 3C–D). Histological analysis of corneal tissue sections further revealed no obvious inflammation or neovascularization in the cornea following the topical placement of the thin films for 7 days (Fig. 3E–G).

### Sunitinib nanofiber thin film prevents corneal neovascularization

**Fig. 4:**
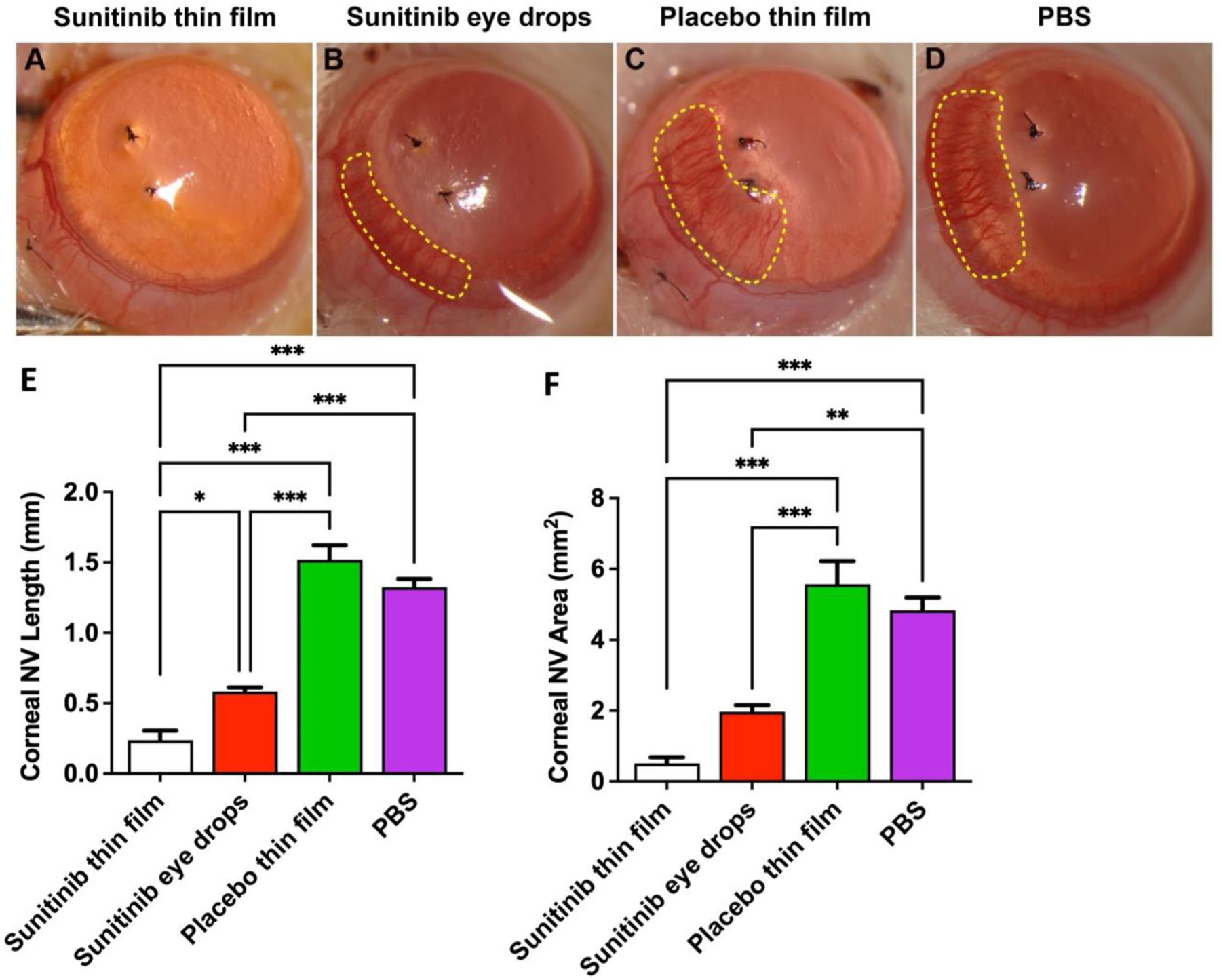
Evaluation of corneal neovascularization 7 days after suture implantation (model induction). Representative images of the suture-induced cornea neovascularization after the treatment of **(A)** sunitinib-eluting thin films (0.15 mg sunitinib, 1 time), **(B)** sunitinib eye drops (5 mg/mL, 10 μL, 3x daily), **(C)** placebo thin films (1 time) and **(D)** PBS (10 μL, 3x daily). Sunitinib-eluting thin films significantly inhibited corneal neovascularization ingrowth **(E)** length and **(F)** area in comparison to PBS, placebo thin films and sunitinib eye drops. Mean ± SEM; *n* = 6 corneas per group; One-way ANOVA followed by Tukey’s multiple comparison test; *p < 0.05; **p < 0.01; ***p < 0.001

A suture-induced rat model of corneal neovascularization was utilized to evaluate the efficacy of sunitinib-eluting thin films. Intrastromal placement of sutures induced new blood vessel invasion into the cornea from the limbus towards the suture stitches by day 7 for the PBS-treated eyes (Fig. 4). Topical application of the placebo thin film showed no inhibition of corneal neovascularization in comparison to PBS-treated eyes. The average corneal NV length for PBS-treated and placebo thin film eyes was 1.29 ± 0.015 and 1.54 ± 0.027 mm, respectively. Topical instillation of 3x daily sunitinib eye drops provided significant improvement in comparison to the placebo thin film (p < 0.001) and PBS (p < 0.001) in inhibiting the ingrowth of corneal NV to 0.62 ± 0.019 mm. The topical placement of sunitinib-eluting thin films for 7 days resulted in significantly increased inhibition and minimal corneal neovascularization, reducing corneal NV ingrowth to 0.29 ± 0.013 mm, in comparison to placebo thin film, PBS, and sunitinib eye drops interventions

The average corneal NV area in the sunitinib thin film group was minimal at 0.61 ± 0.24 mm², representing approximately a 9-fold reduction compared to placebo thin film (5.56 ± 0.65 mm²) (*p* < 0.001) and an 8-fold reduction compared to PBS-treated eyes (4.83 ± 0.36 mm²) (*p* < 0.001). The sunitinib eye drops, administered 3x daily, showed moderate efficacy with an average NV area of 1.97 ± 0.18 mm², which was significantly better than both placebo thin film (*p* < 0.001) and PBS (*p* < 0.01) treatments, but still approximately 3.8-fold greater than the area observed with sunitinib thin film. The placebo thin film showed no inhibitory effect on corneal NV area when compared to PBS-treated controls.

### Sunitinib nanofiber thin film prevents pro-angiogenic cytokine expression

**Fig. 5:**
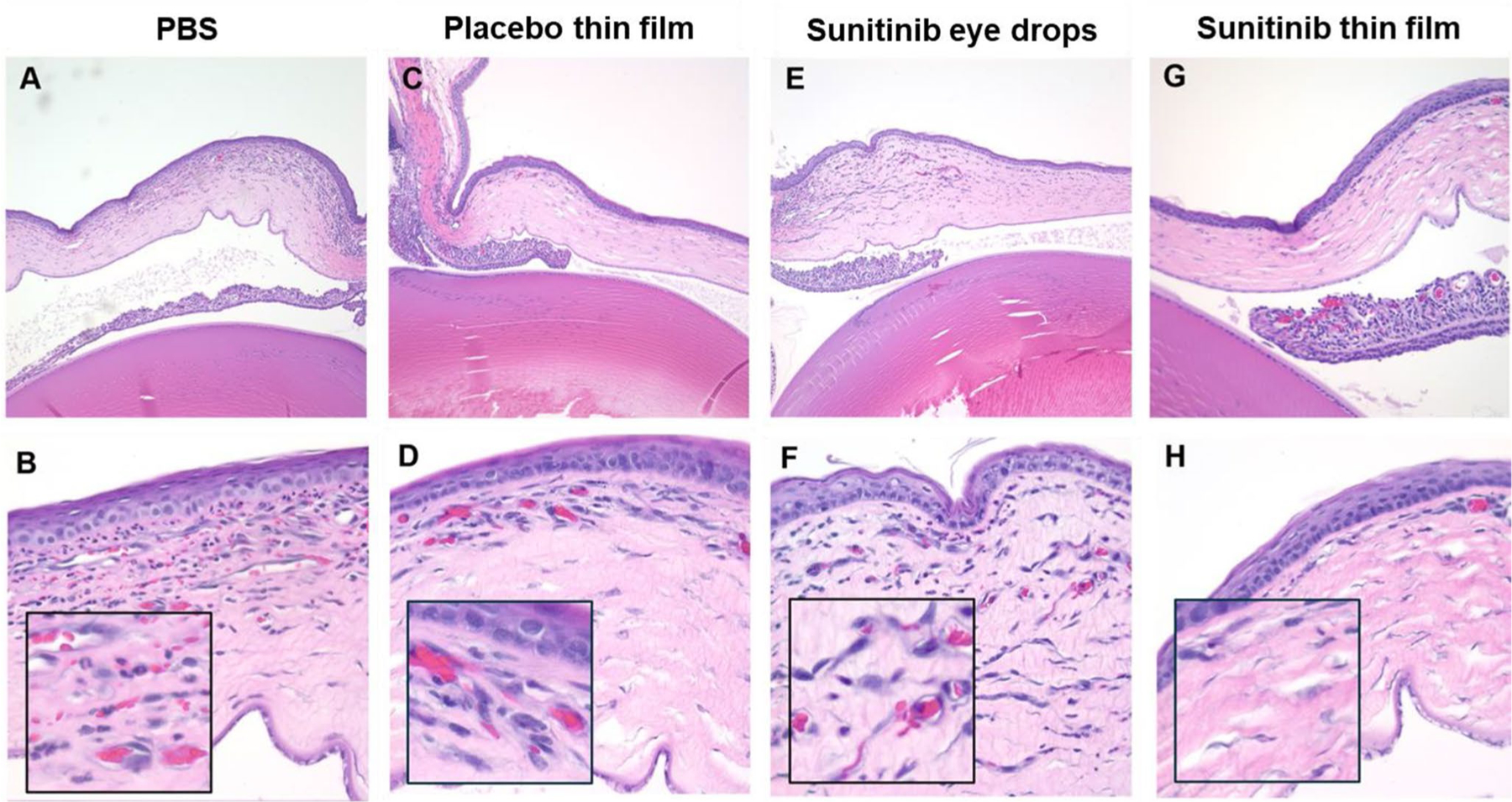
Histological examination of corneas 7 days after model induction and treatment. Extensive neovascularization was observed in eyes receiving **(A**, **B)** PBS (10 μL, 3x daily), **(C**, **D)** placebo thin film (1 time), or **(E**, **F)** sunitinib eye drops (5 mg/mL, 10 μL, 3x daily). **(G**, **H)** Sunitinib thin film (0.15 mg sunitinib, 1 time) treated corneas showed minimal neovascularization. Magnification: top row (100x) and bottom row (400x)

Histopathological analysis confirmed that the intrastromal suture stitches induced extensive corneal neovascularization and corneal inflammation in the PBS, placebo thin film and sunitinib eye drop-treated corneas. However, neovascularization was dramatically inhibited by the one-time topical placement of the sunitinib-eluting thin film (Fig. 5).

**Fig. 6:**
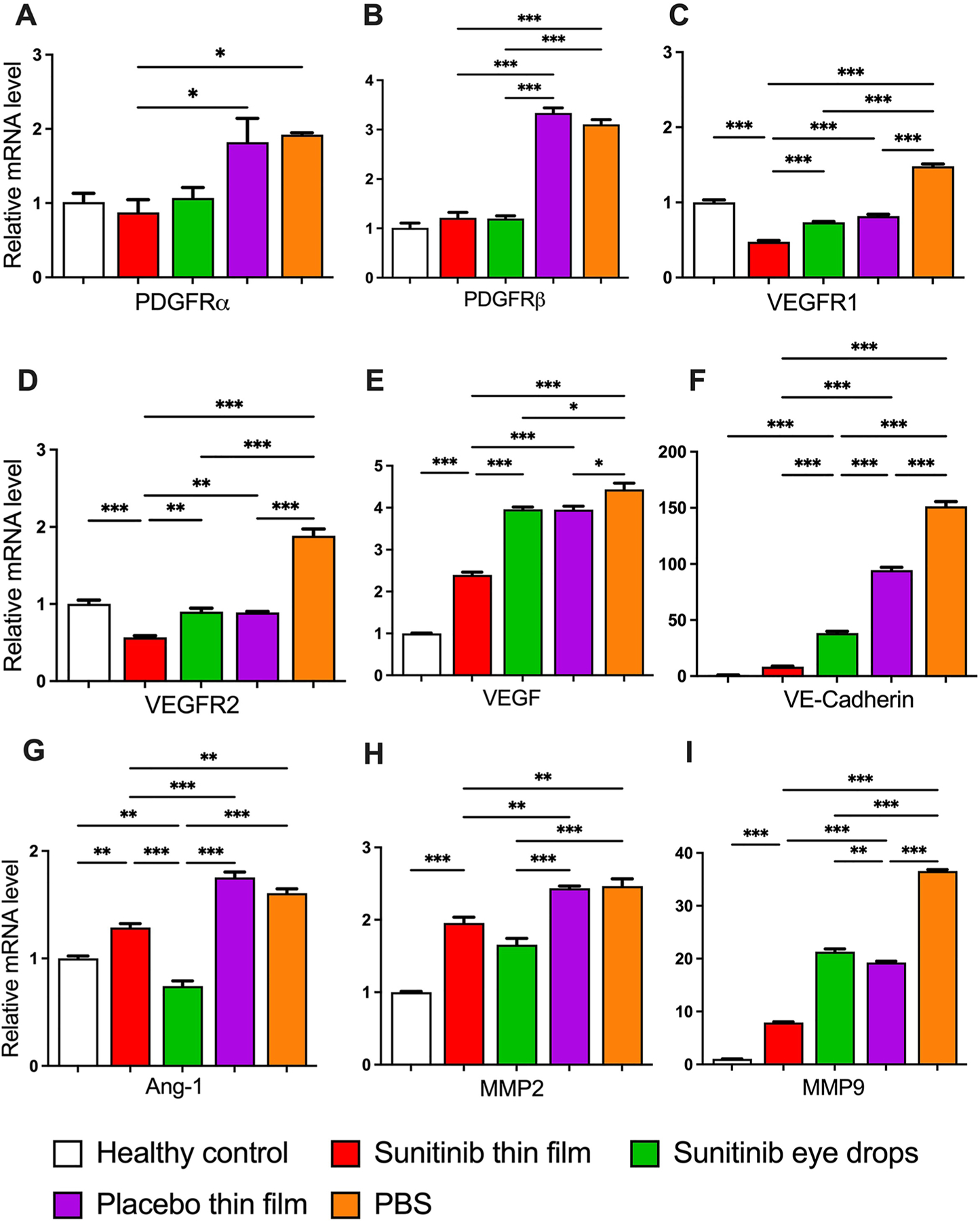
RT-PCR analysis of mRNA expression of **(A)** PDGFRα, **(B)** PDGFRβ, **(C)** VEGFR1, **(D)** VEGFR2, **(E)** VEGF, **(F)** VE-Cadherin, **(G)** Ang-1, **(H)** MMP-2, and **(I)** MMP-9 in the corneas of rats without sutures (healthy) or 7 days after suture placement following administration of sunitinib thin film (0.15 mg sunitinib, 1 time), topical sunitinib eye drops (5 mg/mL, 10 μL, 3x daily), placebo thin film (1 time), or PBS (10 μL, 3x daily). Mean ± SEM; *n* = 3 repeats from the pooled 3 corneas of the same condition; One-way ANOVA followed by Tukey’s multiple comparison test; *p < 0.05; **p < 0.01; ***p < 0.001

Topical application of the sunitinib-eluting thin film significantly downregulated the gene expression of PDGFRs and VEGFRs in addition to angiogenesis-related cytokines such as MMP2, MMP9, Ang-1, VE-cadherin, in comparison to PBS, placebo thin film and sunitinib eye drop-treated corneas (Fig. 6). In brief, compared to healthy controls, the sunitinib thin film maintained near-normal expression levels of critical growth factor receptors like PDGFRα and PDGFRβ; showing minimal change while placebo and PBS groups exhibited significant 1.8-fold and 3-fold increases, respectively. The sunitinib thin film’s protective effect was particularly evident for VEGFR1 and VEGFR2, where it not only prevented upregulation but actually reduced VEGFR2 expression to approximately 50% of control levels, while PBS treatment caused nearly 2-fold increases in both receptors. For pro-angiogenic factors, the sunitinib thin film demonstrated superior inhibitory effects compared to all other treatments. While PBS treatment led to ~ 150-fold increase in VE-Cadherin and ~ 4.5-fold rise in VEGF, sunitinib thin film significantly reduced their expression, maintaining VE-Cadherin nearly 11-fold and 18-fold lower than in placebo and PBS-treated eyes, respectively. Similarly, VEGF was about 2-fold lower than in placebo- and PBS treated eyes. The suppression of matrix metalloproteinase upregulation was particularly striking. Sunitinib thin film restricted MMP2 elevation to ~ 2-fold, compared to ~ 2.5-fold in both PBS and placebo groups. Similarly, MMP9 expression was restricted to ~ 8-fold, whereas PBS and placebo treatment led to a dramatic ~ 37-fold and ~ 20-fold increase compared to healthy controls, respectively. Minimal differences in angiogenic gene expression were observed between placebo film and PBS groups, confirming that the PLGA film alone has negligible biological activity.

## Discussion

There is a significant and unmet need for novel prophylactic and therapeutic modalities for corneal neovascularization that are sustained, highly effective, and biocompatible. Here, we designed, manufactured, and evaluated a novel sunitinib-eluting thin film for prevention of corneal neovascularization. In order to provide clinical utility, thin films must provide appropriate strength for topical application and retention, sustained drug release of an effective anti-angiogenic agent, and suitable diffusion of oxygen to the cornea. Nanofiber and microfiber-based films were manufactured through modification of electrospinning and formulation parameters, such as PLGA wt%, flow rate, needle gauge, applied voltage, and distance to collector. Increased wt%, flow rate, and needle inner diameter combined with decreased voltage and collector distance allowed for reproducible manufacture of microfiber films. Microfiber films demonstrated superior tensile strength and permeability and demonstrated linear in vitro drug release for at least 30 days. However, nanofiber thin films provided increased drug delivery functionality in the early post-operative period, and provided sufficient strength and permeability, as shown by fluorescein staining of rat corneas following thin film application. Nanofiber-based sunitinib thin films inhibited corneal neovascularization for at least 7 days in a suture-induced rat model of angiogenesis.

Disorders of the cornea and insults to the cornea lead to upregulation of one or more pro-angiogenic signaling molecules, including VEGF, PDGF, MMP, interleukin-1, and basic fibroblast growth factor [7]. Sunitinib is a particularly promising candidate for prevention and/or treatment of corneal neovascularization due to its multi-targeted inhibitory activity, which has been shown to be more effective than anti-VEGF biologics [4, 27, 28]. This study revealed the potential for sunitinib to broadly inhibit corneal neovascularization, regardless of the pathological cause or angiogenic pathway. Sunitinib-eluting thin films successfully down-regulated mRNA expression of PDGFRα, PDGFRβ, VEGFR1, VEGFR2, VEGF, VE-Cadherin, and MMP-9. Notably, each of these markers was down-regulated more significantly by sunitinib-eluting thin films than by 3x daily sunitinib eye drops, except for PDGFs. Sunitinib-eluting thin films reduced neovascularization length and area by more than 50% in comparison to topical eye drops, demonstrating the benefit of local, long-term drug delivery to the cornea. Through a minimally invasive approach, thin films are able to address many of the challenges with topical ocular drug delivery, including patient compliance, the barrier properties of the cornea, and tear dilution and turnover [29].

Importantly, electrospun thin films provide a highly versatile approach to ocular drug delivery. Recent reports have shown the utility of electrospun films for tunable delivery of anti-fungal, anti-infective, anti-angiogenic, and steroidal agents ranging from hours to months [30–33]. Film characteristics, such as drug loading, degradation rate, thickness, porosity, and fiber diameter can be reproducibly tuned by modifying polymer/drug formulation and manufacturing parameters to modulate mechanical properties, permeability, and duration and rate of drug release [11]. These thin films are compatible with a wide range of therapeutic agents with varying physicochemical properties, including small molecule drugs, biologics, and genes. While ex vivo sunitinib permeation was not assessed in this study it will be included in future pharmacokinetic analyses along with other classes of therapeutic moieties. Notably, thin films can be loaded with multiple drugs with different mechanisms of action to further improve clinical outcomes. For example, thin films could be loaded with multiple anti-angiogenic molecules to improve treatment of severe corneal neovascularization characterized by mature vessels. Alternatively, thin films could simultaneously deliver antibiotics and anti-angiogenic agents to address major causes of penetrating keratoplasty rejection and failure [2, 3, 34, 35]. Sunitinib-eluting thin films have potential to not only block pro-angiogenic pathways but also stop blood vessel growth and regress existing vessels. Although this study examined prevention of corneal neovascularization, future studies will focus on treatment of corneal neovascularization and considerations for clinical translation, including film application, positioning, and degradation.

### Future perspectives

Future work will address translational aspects including film retention without sutures, long-term in vivo pharmacokinetics and degradation, including in an inflammatory setting, and adaptation for postoperative use in keratoplasty. Development of films with dual anti-angiogenic and anti-infective agents may further enhance clinical utility. Additionally, optimized film geometries and adhesive coatings are under investigation to enable suture-free placement.

## Conclusions

Corneal neovascularization is a leading cause of visual impairment and blindness that requires new treatment modalities to improve visual outcomes and reduce the risk of corneal transplant failure. This study demonstrated the manufacture and evaluation of biodegradable, sunitinibeluting nano- and micro-fiber thin films designed to inhibit corneal neovascularization via topical administration. In vitro experiments revealed that both films provided appropriate tensile strength and permeability, and sustained sunitinib release for at least 30 days. In vivo experiments revealed that application of nanofiber-based thin films to the rat cornea for 7 days did not lead to inflammation, ischemia, or epithelial damage. Importantly, sunitinib-eluting thin films significantly down-regulated gene expression of multiple pro-angiogenic factors, including PDGFRs, VEGFRs, MMP2, MMP9, Ang-1, and VE-cadherin, resulting in significantly greater inhibition of suture-induced corneal neovascularization than 3x daily application of sunitinib eye drops. This versatile, multi-targeted sustained drug-delivery platform has potential to provide a safe and highly effective treatment for corneal or other ocular surface disorders.

## Supplementary Material

Supplementary Materials

**Supplementary Information** The online version contains supplementary material available at https://doi.org/10.1007/s13346-025-01926-5.

## Figures and Tables

**Table 1:** Thickness, fiber diameter, permeability, and breaking strength of PLGA and PLGA/sunitinib thin films. Values expressed as mean ± sem

	Thin film	Spray time (min)	Thickness (μm)	Fiber diameter	Water vapor flux (g/(h·m^2^))	Breaking strength (*N*)
Nanofiber	PLGA	120	13 ± 2	320 ± 20 nm	186 ± 7	0.64 ± 0.02
	PLGA/Sunitinib	120	13 ± 2	350 ± 30 nm	175 ± 6	0.69 ± 0.06
Microfiber	PLGA	20	43 ± 2	3.2 ± 0.3 μm	218 ± 18	1.08 ± 0.18
	PLGA/Sunitinib	20	46 ± 5	4.5 ± 0.4 μm	214 ± 24	1.04 ± 0.27

## Data Availability

The datasets generated during and/or analyzed during the current study are available from the corresponding author on reasonable request.
