# Supplementary material for: Sunitinib-eluting thin films for Inhibition of corneal neovascularization
Source: Drug Deliv Transl Res. 2025 Jul 28;16(4):1023–34. doi: 10.1007/s13346-025-01926-5 (PMC12777655; doi:10.1007/s13346-025-01926-5)
Supplement: Supplementary file 1 — Supplementary Material 1 [file 13346_2025_1926_MOESM1_ESM.pdf]

Supplementary Materials

Sunitinib-eluting thin films for inhibition of corneal neovascularization

Kunal S. Parikh [1,2,3,4‡], Jin Yang [1,2,5‡], Zheng Ding [1,4], Richard Shi [1,4], Sagun Poudel [6], Yumin Oh [1,2], Lixia Luo [1,2,7], Shiyu Xia [1,8], Gregg Duncan [1,2,9], Charles Eberhart [2,10], Laura M. Ensign [1,2,4,8], Justin Hanes [1,2,4,8], Qingguo Xu [1,2,6,11,*]

[1] Center for Nanomedicine, The Wilmer Eye Institute, Johns Hopkins University School of Medicine, Baltimore, MD 21231, USA

[2] Department of Ophthalmology, The Wilmer Eye Institute, Johns Hopkins University School of Medicine, Baltimore, MD 21287, USA

[3] Center for Bioengineering Innovation & Design, Johns Hopkins University, Baltimore, MD 21218, USA

[4] Department of Biomedical Engineering, Johns Hopkins University School of Medicine, Baltimore, MD 21205, USA

[5] Department of Ophthalmology, Myopia Key Laboratory of Health PR, Eye & ENT Hospital of Fudan University, Shanghai, 200031, China

[6] Departments of Pharmaceutics, Virginia Commonwealth University, Richmond, VA 23298, USA

[7] State Key Laboratory of Ophthalmology, Zhongshan Ophthalmic Center, Sun Yat-sen University, Guangzhou, 510060, China

[8] Department of Chemical and Biomolecular Engineering, The Johns Hopkins University, Baltimore, MD 21218, USA

[9] Fischell Department of Bioengineering, University of Maryland, College Park, MD 20742

[10] Department of Pathology, The Johns Hopkins University School of Medicine, Baltimore, MD 21231, USA

[11] Departments of Ophthalmology, Pediatrics, Biomedical Engineering, Massey Cancer Center, and Center for Pharmaceutical Engineering, Virginia Commonwealth University, Richmond, VA 23298, USA

‡ These authors contributed equally.
* To whom correspondence should be addressed: qxu@vcu.edu

Table S1. RT-PCR primer sequences

	Primer sequence
**GAPDH**	F: ATGCTGGTGCTGAGTATGTC
	R: AGTTGTCATATTTCTCGTGG
**VEGFR1**	F: CGTGAAGCATCGGAAGCAA
	R: ACCGAATAGCGAGCAGATTTCT
**VEGFR2**	F: TTTGGCAAATACAACCCTTCAGAT
	R: ACTCTGGGA ACTGTGAGTGTCTTG
**PDGFR-a**	F: CGACTCCAGATGGGAGTTCCC
	R: TGCCATCCACTTCACAGGCA
**PDGFR-b**	F: CAACATTTCGAGCACCTTTGT
	R: AGGGCACTCCGAAGAGGTAA
**VEGF**	F：GCCCATGAAGTGGTGAAGTT
	R：ACTCCAGGGCTTCATCATTG
**VE-Cadherin**	F: CCAGAATTTGCCCAGCCCTA
	R: GTCCTCGTTCTTCAGGGCAA
**Ang-1**	F: ACGGGAACTGCCGTGAGA
	R: CACGTGTGGAGCATCCCTTT
**MMP-2**	F: AGCTTTGATGGCCCCTATCT
	R: GGAGTGACAGGTCCCAGTGT
**MMP-9**	F: CCACCGAGCTATCCACTCAT
	R: GTCCGGTTTCAGCATGTTTT

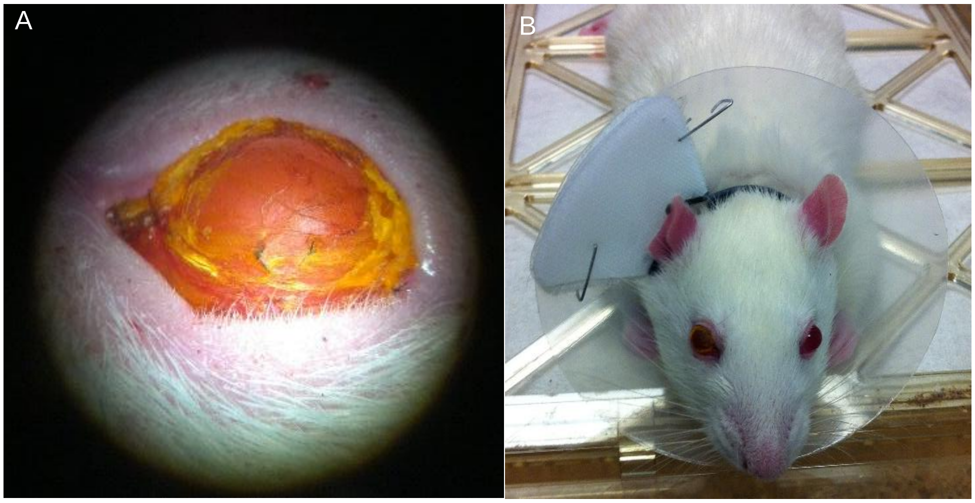

Figure S1. Application of the sunitinib thin film to the rat ocular surface. (A) The image shows how the thin film was applied to the cornea with suture-induced corneal neovascularization. (B) An Elizabeth cone was applied after thin film placement.
